# Epistatic Gene-Based Interaction Analyses for Glaucoma in eMERGE and NEIGHBOR Consortium

**DOI:** 10.1371/journal.pgen.1006186

**Published:** 2016-09-13

**Authors:** Shefali Setia Verma, Jessica N. Cooke Bailey, Anastasia Lucas, Yuki Bradford, James G. Linneman, Michael A. Hauser, Louis R. Pasquale, Peggy L. Peissig, Murray H. Brilliant, Catherine A. McCarty, Jonathan L. Haines, Janey L. Wiggs, Tamara R. Vrabec, Gerard Tromp, Marylyn D. Ritchie, eMERGE Network, NEIGHBOR Consortium

**Affiliations:** 1 Department of Biomedical and Translational Informatics, Geisinger Health System, Danville, Pennsylvania, United States of America; 2 The Huck Institute of Life Sciences, The Pennsylvania State University, University Park, Pennsylvania, United States of America; 3 Department of Epidemiology and Biostatistics, Institute for Computational Biology, Case Western Reserve University School of Medicine, Cleveland, Ohio, United States of America; 4 Center for Human Genetics, Marshfield Clinic Research Foundation, Marshfield, Wisconsin, United States of America; 5 Department of Ophthalmology, Duke University Medical Center, Durham, North Carolina, United States of America; 6 Department of Ophthalmology, Harvard Medical School, Massachusetts Eye and Ear Infirmary, Boston, Massachusetts, United States of America; 7 Channing Division of Network Medicine, Brigham and Women's Hospital, Harvard Medical School, Boston, Massachusetts, United States of America; 8 Essentia Rural Health, Duluth, Minnesota, United States of America; 9 Department of Ophthalmology, Geisinger Health System, Danville, Pennsylvania, United States of America; 10 Division of Molecular Biology and Human Genetics, Department of Biomedical Sciences, Faculty of Medicine and Health Sciences, Stellenbosch University, Tygerberg, South Africa; 11 Department of Biochemistry and Molecular Biology, The Pennsylvania State University, University Park, Pennsylvania, United States of America; University of Miami, Miller School of Medicine, UNITED STATES

## Abstract

Primary open angle glaucoma (POAG) is a complex disease and is one of the major leading causes of blindness worldwide. Genome-wide association studies have successfully identified several common variants associated with glaucoma; however, most of these variants only explain a small proportion of the genetic risk. Apart from the standard approach to identify main effects of variants across the genome, it is believed that gene-gene interactions can help elucidate part of the missing heritability by allowing for the test of interactions between genetic variants to mimic the complex nature of biology. To explain the etiology of glaucoma, we first performed a genome-wide association study (GWAS) on glaucoma case-control samples obtained from electronic medical records (EMR) to establish the utility of EMR data in detecting non-spurious and relevant associations; this analysis was aimed at confirming already known associations with glaucoma and validating the EMR derived glaucoma phenotype. Our findings from GWAS suggest consistent evidence of several known associations in POAG. We then performed an interaction analysis for variants found to be marginally associated with glaucoma (SNPs with main effect p-value <0.01) and observed interesting findings in the electronic MEdical Records and GEnomics Network (eMERGE) network dataset. Genes from the top epistatic interactions from eMERGE data (Likelihood Ratio Test i.e. LRT p-value <1e-05) were then tested for replication in the NEIGHBOR consortium dataset. To replicate our findings, we performed a gene-based SNP-SNP interaction analysis in NEIGHBOR and observed significant gene-gene interactions (p-value <0.001) among the top 17 gene-gene models identified in the discovery phase. Variants from gene-gene interaction analysis that we found to be associated with POAG explain 3.5% of additional genetic variance in eMERGE dataset above what is explained by the SNPs in genes that are replicated from previous GWAS studies (which was only 2.1% variance explained in eMERGE dataset); in the NEIGHBOR dataset, adding replicated SNPs from gene-gene interaction analysis explain 3.4% of total variance whereas GWAS SNPs alone explain only 2.8% of variance. Exploring gene-gene interactions may provide additional insights into many complex traits when explored in properly designed and powered association studies.

## Author Summary

The complex nature of primary-open angle glaucoma (POAG) has left researchers exploring the genetic architecture and searching for the missing heritability using a number of different study designs. Over the past decade, many studies have been conducted to explain the etiology of POAG; however, a high proportion of estimated heritability still remains unexplained. GWA studies for POAG have identified significant associations but these associations have only explained a small proportion of the genetic risk (odds ratios range between 1–3). In this paper, we sought to confirm the primary genome-wide significant associations that have been discovered so far for glaucoma in phenotypes developed from EMR data in an effort to show that EMR data can be a powerful resource for finding genetic variants influencing POAG susceptibility. Next, we tested for statistical interactions, which can be presented as an important tool in an attempt to explain POAG heritability. We used a reduced list of variants filtered by marginal main effect analysis to look for epistatic interactions. We present our results from replication of gene-based interaction analyses performed in eMERGE and the NEIGHBOR consortium data. Using expression data and annotations from various publicly available databases, the most significant genes that replicated in our analyses show expression in the eye and trabecular meshwork. Analysis for estimation of genetic variance explained by significant associations from previous GWAS and replicated variants from gene-based interactions suggest that these explain 5.6% of variance in eMERGE dataset and also explain 3.4% variance in NEIGHBOR dataset.

## Introduction

Glaucoma, a chronic degenerative optic neuropathy that results in loss of retinal ganglion cells and axons, is one of the primary causes of irreversible visual impairment worldwide affecting approximately 70 million people[1]. Twin and relative studies have estimated the heritability of primary open angle glaucoma (POAG) to be between 16–20%[2,3]. Genetic linkage studies have only identified a common mutation found in *MYOC* which explains a very small fraction of total risk in different populations[4]. Genome-wide association studies (GWAS) have proven to be a successful tool in identifying several loci and genes associated with POAG which have elucidated important biological information that has enhanced some understanding of the genetic architecture of this disease[5–8]. Family based studies and GWAS taken together still only explain less than 10% of the heritability of POAG[9].

Phenotypes from electronic medical records (EMR) data are curated and linked to genetic bio-repositories in the eMERGE (electronic MEdical Records and GEnomics) network at several participating institutions nationwide[10–12]. In eMERGE, genotypes in more than 55,000 samples from 9 sites and 9 different genotyping platforms have been imputed to the 1000 Genomes reference panel[13]. We conducted a GWAS on 5,090 eMERGE samples (961 cases and 4,129 controls) that were extracted from the dataset containing ~55,000 samples for the purpose of confirming known signals from previous POAG GWAS to show that EMRs are a powerful resource that can be utilized to explore genetic associations for complex traits. In addition to disease- associated SNPs identified by GWAS, some studies have also identified interactions between variants in genes *OPTN* and *OLFM2* to be significantly associated with open angle glaucoma (OAG)[14]. The genetic architecture of traits with complex inheritance such as POAG are expected to include complex genetic interactions, that in part, can also account for disease heritability [15–19]. Many studies in model organisms such as drosophila and mouse have shown strong evidence of epistasis, or gene-gene interactions, in complex phenotypes [20,21]. Because of the complex nature of POAG, it is possible that disease risk is not mediated only by single loci but also by a harmonious links between different genes.

Many computational techniques have emerged in recent years to explore genetic interactions, yet numerous challenges must be overcome to conduct these analyses, including extensive computational resources and time required to run analyses. Further, exhaustively searching for pairwise SNP-SNP interactions leads to issues with multiple testing corrections, which makes discovering interactions even more difficult. To limit the search space, it is important to reduce the number of variants being tested[22]. To address this issue, we limited our SNP-SNP interaction analysis in the eMERGE dataset (Discovery dataset) to only those SNPs attaining marginal to strong association (p-value < 0.01) in the eMERGE POAG GWAS.

Additional analytic challenges include biological interpretation of all SNP-SNP interactions. Replication of the exact SNP pairs is extremely difficult and still remains an exasperating task as it is complicated by linkage disequilibrium (LD) pattern differences as well as genetic and/or clinical heterogeneity [23–25]. Genetic heterogeneity possesses a challenge in replicating exact variants associated with a disease in different populations, as the variants are spread across multiple loci in a gene or are in high linkage disequilibrium (LD) with other variants. It has been previously reported from studies in model organisms such as yeast that understanding the genetic architecture of diseases precisely in heterogeneous populations such as humans is an arduous task [26–28]. Therefore, to overcome the problem of replicating exact SNP-SNP pairs and address the challenge of heterogeneity, we annotated SNPs to genes and then extracted all SNPs in the top interacting models (results with likelihood ratio test (LRT) p-value<1e-05 in the Discovery dataset) to perform a gene-based SNP-SNP interaction analyses followed by replication in the NEIGHBOR consortium (Replication dataset; previously genotyped and reported) [29]. The dataset, which includes 2,132 POAG cases and 2,290 non-POAG controls, was then imputed to the 1000Genomes reference dataset[30]. In this study, we report our top replicated findings from the first gene-based interaction study in POAG conducted on two independent datasets.

## Results

### Generalization results from GWAS

**Fig 1:**
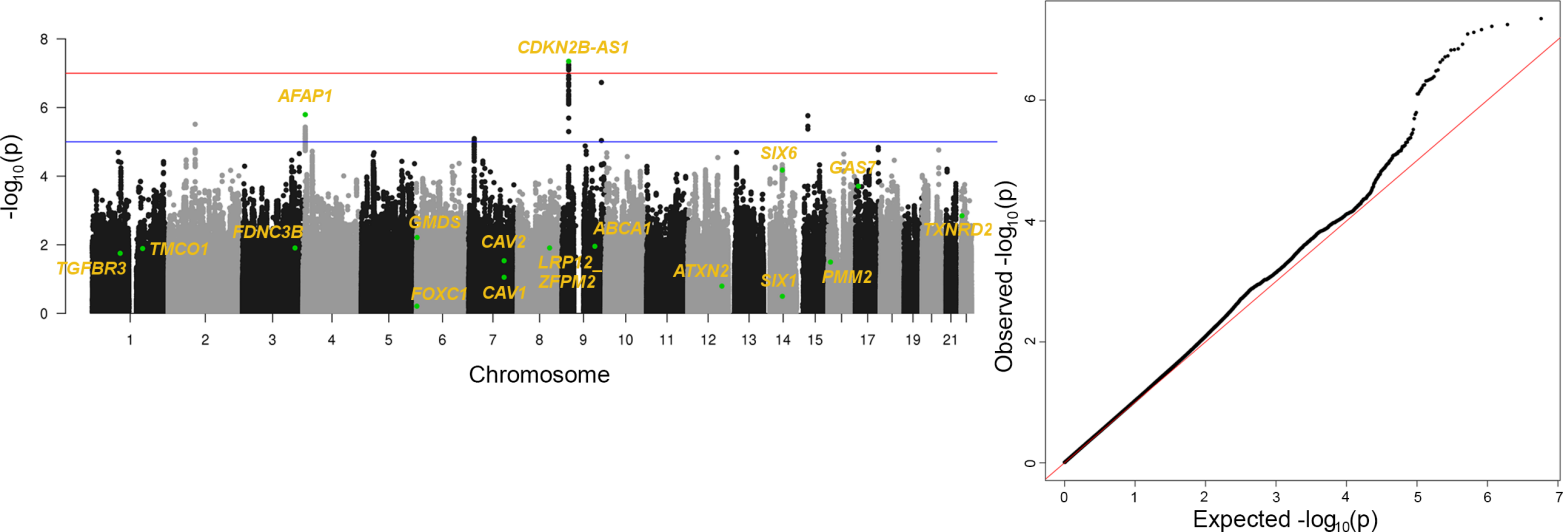
POAG GWAS Results in eMERGE dataset. **(A)** Manhattan plot shows chromosome and base pair positions on x-axis and -log10 of p-value on y-axis. Each point here represents a SNP. Red line is the genome-wide significance line at 5e-08. Highlighted points indicate annotation of SNPs with previously reported GWAS associated genes. **(B)** Shows a QQ-plot from GWAS analysis.

To identify previously reported associations between SNPs and POAG disease status as defined by the EMR algorithm, we performed a genome-wide scan with samples classified as POAG cases (N = 961) and controls (N = 4,129). The POAG cases were electronically phenotyped using the EMR data in eMERGE (details on the phenotypic algorithm for POAG are provided in supplementary material S1 Text). Our analysis was adjusted for the first six principal components, age, sex, genotyping platform, and eMERGE site. **Fig 1A** illustrates the results for the primary GWAS analysis in a Manhattan plot. SNPs in genes that have known association with glaucoma were annotated with gene names for simplicity of interpretation of results. **Fig 1B** shows a quantile-quantile plot of observed and expected p-values. The genomic inflation factor (λ) is 1.04, which suggests that there is minimal inflation in our results.

From the GWAS analysis, we reviewed 17 previously reported glaucoma-associated loci that have attained genome-wide significance (i.e. p-values<5e-08) according to the NHGRI GWAS catalog in one or more studies[31]. **Table 1**shows all of the known loci as of March 25, 2016 and with the p-values obtained from our analyses. Although only one of our results reaches genome-wide significance level (5e-08), six of our results reach Bonferroni significance (0.05/17 tests), and 13/17 exceed a p-value <0.05; thus, we observe p-values for known associations also clearly shows that EMR data can produce non-spurious and relevant findings. Thus, we believe that the data derived from the EMR were able to replicate many of the signals and demonstrate that in our EHR-derived phenotype we can identify association signals with glaucoma. A point to note here is that based on previous GWAS’s as cited from NHGRI GWAS catalog, not all loci reach genome-wide significance in all studies. Here we have compiled lists of all loci that have reached genome-wide significance in a minimum of 1 study. For example, variants in gene *PMM2* have so far only being replicated in Chinese populations. Based on power calculations, we had approximately 45% power to detect a common allele with odds ratio greater than 1.5 at genome wide significance (S1 Table shows power calculation results considering odds ratio in range on 1–3 as reported in previous GWAS studies for POAG in the NHGRI GWAS catalog and MAF range of 0.05–0.5). Power calculations were done using Quanto available at: http://biostats.usc.edu/Quanto.html. Thus, we demonstrate that existing EMR data can be extensively utilized in confirming known GWAS hits and are powerful resources for applying new methodologies to explore the genetic architecture of complex traits.

**Table 1:** Results from GWAS analysis, showing previously reported gene regions that are known to be associated with glaucoma; the lowest p-value of the gene region in eMERGE GWAS analysis for glaucoma is reported.

*Gene Region*	Chromosome	Start Position	End Position	SNP	Lowest P-value	Odds Ratio	Reference Study
*CDKN2B-AS*	9	21984777	22131096	rs7866783	4.49E-08	1.34	[57]Osman W (PMID: 22419738)
*AFAP1*	4	7750440	7951653	rs1320074	1.61E-06	0.77	[5]Gharahkhani P (PMID: 25173105)
*SIX6*	14	60965669	60989568	rs1018533	6.65E-05	0.80	[5]Gharahkhani P (PMID: 25173105)
							[57]Osman W (PMID: 22419738)
*GAS7*	17	9803926	10111868	rs12150284	1.95E-04	1.22	[58]van Koolwijk LM (PMID: 22570627)
*TXNRD2*	22	19853040	19939359	rs58714937	1.43E-03	1.26	[30]Cooke Bailey J (PMID: 26752265)
*GMDS*	6	1614035	2255868	rs13217030	6.11E-03	1.21	[5]Gharahkhani P (PMID: 25173105)
*ABCA1*	9	107533283	107790527	rs2472495	1.10E-02	1.17	[45]Chen Y (PMID: 25173107)
*FNDC3B*	3	171747418	172128493	rs12897	1.24E-02	0.87	[32]Lu Y(PMID: 23291589)
*8q22 Region*: *LRP12_ZFPM2*	8	105491459	106826767	rs1545699	1.23E-02	0.86	[29]Wiggs J (PMID: 22570617)
*TMCO1*	1	165683528	165806992	rs4233408	1.28E-02	0.82	[58]van Koolwijk LM (PMID: 22570627)
							[5]Gharahkhani P (PMID: 25173105)
*TGFBR3*	1	92135900	92381559	rs2810903	1.77E-02	1.24	[33]Li Z (PMID: 25861811)
*PMM2*	16	8890670	8944194	rs8057024	3.18E-02	1.11	[45]Chen Y (PMID: 25173107)
*CAV2*	7	115917434	116158595	rs75347112	2.91E-02	0.79	[7]Thorleifsson G (PMID: 20835238)
*CAV1*	7	116154839	116211239	rs7795510	8.84E-02	1.09	[7]Thorleifsson G (PMID: 20835238)
*ATXN2*	12	111880018	112047480	rs653178	1.60E-01	1.08	[30]Cooke Bailey J (PMID: 26752265)
*SIX1*	14	61100133	61134977	rs7152548	3.17E-01	0.90	[57]Osman W (PMID: 22419738)
*FOXC1*	6	1600681	1624132	rs2235718	6.15E-01	0.96	[30] Cooke Bailey J (PMID: 26752265)

### Gene based SNP-SNP Interactions

**Fig 2:**
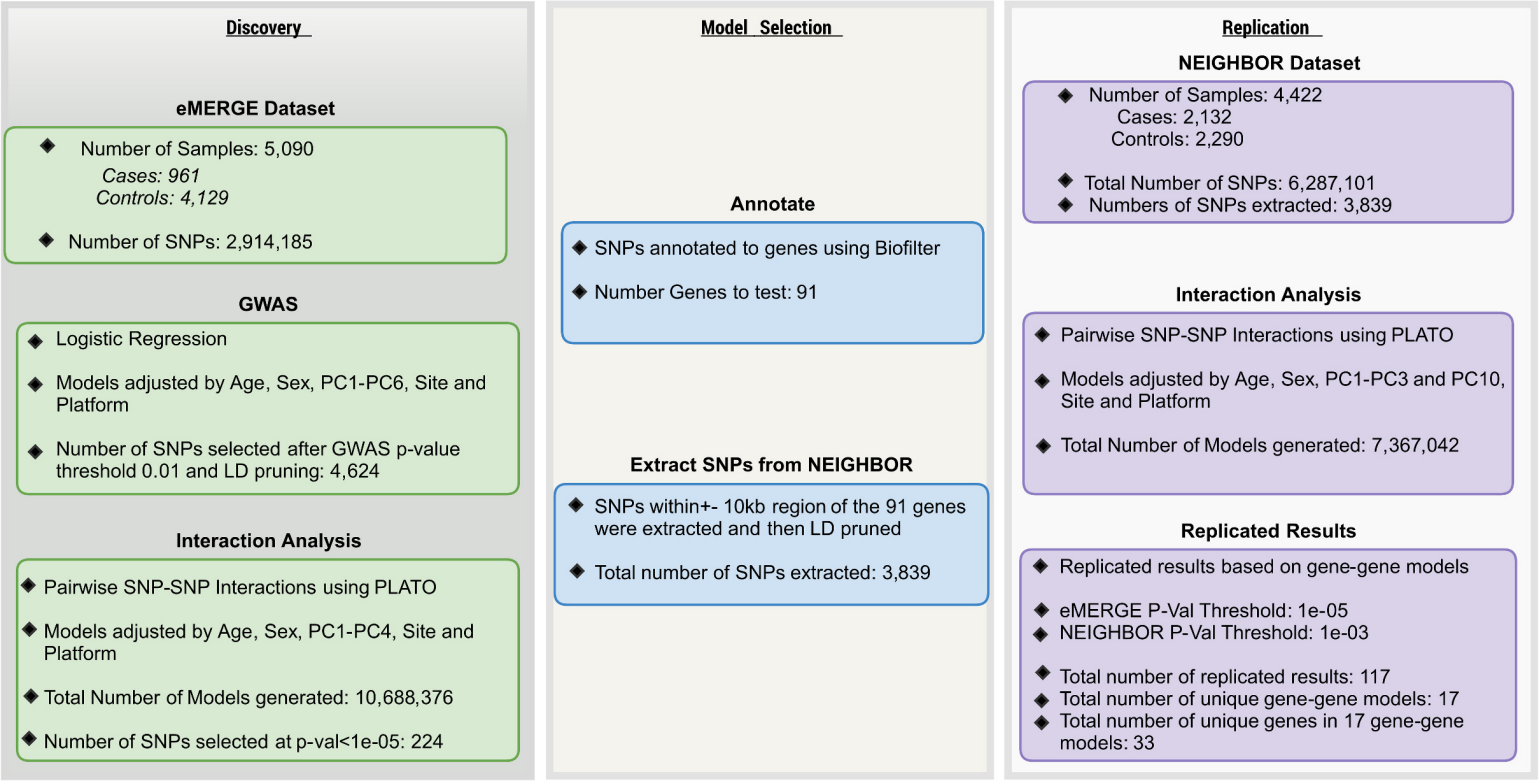
Flow chart explaining all the steps for gene based interaction analysis in discovery (eMERGE) and replication dataset (NEIGHBOR).

**Fig 3:**
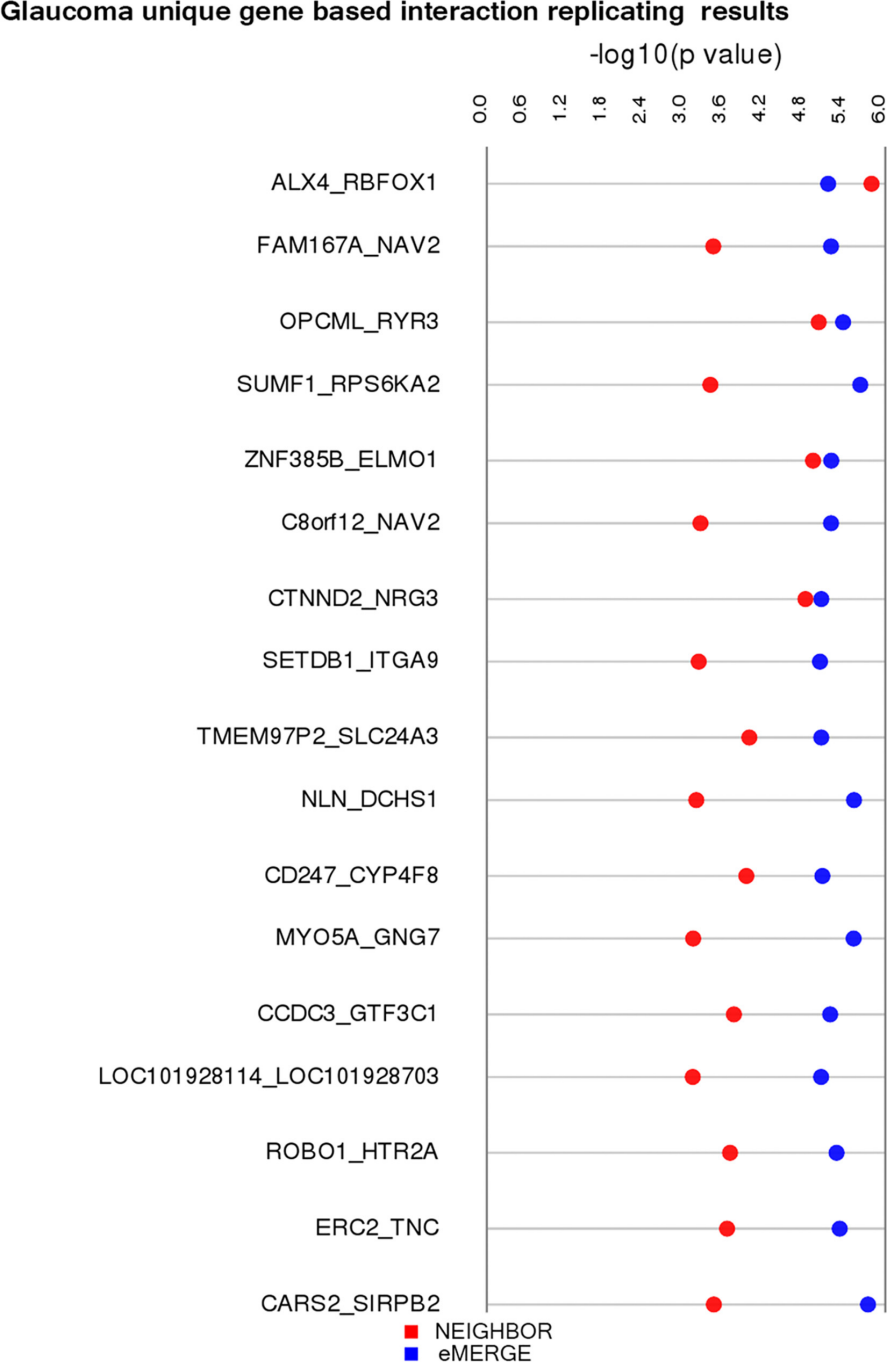
Synthesis view plot representing unique gene-gene interaction models and their p-values in discovery and replication dataset.

Detection of epistasis is of interest in exploring the etiology of complex diseases. In the present study, markers that showed suggestive marginal association with POAG from the eMERGE GWAS (4,624 SNPs with p-value<0.01) were chosen as potential candidates to detect epistatic interactions between genes, a strategy also used by many others (reviewed by Sun et al 2014) [32]. We performed this analysis in all 5,090 eMERGE samples and also in samples only from European American (EA) ancestry (4,840 samples). In testing for epistasis, we performed a pairwise SNP-SNP interaction analysis i.e. each from the 4,624 SNPs were tested against the remaining 4,623 SNPs. To examine the distribution of observed p-values in this discovery dataset, we plotted the p-values in a quantile-quantile plot to compare against random expected p-values. This qq-plot is shown in S1 Fig. We also calculated the inflation factor for the observed values using the GenABEL package in R[34] and the inflation factor was 1.03. SNPs within or 10kb upstream or downstream of the gene location were mapped to 2,433 genes using the Library of Knowledge Integration (LOKI) database compiler in Biofilter 2.0[33]. From the eMERGE dataset, we chose a p-value threshold (1e-05) to obtain approximately the top 100 results to consider for replication. Next, to empirically estimate the null distribution of the test statistics in the discovery dataset, we conducted the Kolmogorov-Smirnov test for all p-values above the chosen threshold (1e-05) to test the null hypothesis that p-values come from a uniform distribution. The test resulted in a p-value of 0.28 which suggests that we did not have proper evidence to reject the null hypothesis. Using a LRT p-value <1e-05 as a threshold, we observed 117 SNP-SNP models that mapped to 91 genes. SNPs (151 out of 224) not located within 10Kb region of a gene were not tested in replication based on the definition of a gene as described above. A total of 3,839 SNPs were available in the NEIGHBOR dataset that mapped to the 91 genes from eMERGE and were subsequently moved forward to the replication stage. There were 1,361 results that passed p-value criteria for marginal replication i.e. LRT p-value <0.01 in the NEIGHBOR dataset (S2 Table). Considering LRT p-value <0.001 (to get the top 100 models) for the NEIGHBOR dataset, we observed 117 SNP-SNP models in 33 unique genes that replicated consistently with the eMERGE results. The flow of this process is explained in detail in **Fig 2**. Since we tested 91 unique genes in NEIGHBOR dataset, we calculated Bonferroni significance at alpha = 0.05 based on these 91 genes (i.e. 0.05/4095 = 1.2E-05 where 4095 is the number of tests for 91 genes), 3 models (*ALX4-RBFOX1*, *OPCML-RYR3 and ZNF385B-ELMO1*) passed Bonferroni corrected p-value (1.2E-05) at alpha = 0.05. **Fig 3**illustrates all unique gene-gene models (resulted in 17 unique models from a total of 117 SNP-SNP non-unique models) and their respective lowest p-values from the models in both eMERGE and NEIGHBOR datasets. Supplementary S2 Table shows all non-unique results that were replicated from both all eMERGE samples as well as only EA eMERGE samples. It is evident from the table that many tag SNPs spread across the same genes show similar interactions, supporting the approach of gene-based interaction analysis rather than finding exact SNP-SNP interaction in both discovery and replication analyses.

**Fig 4:**
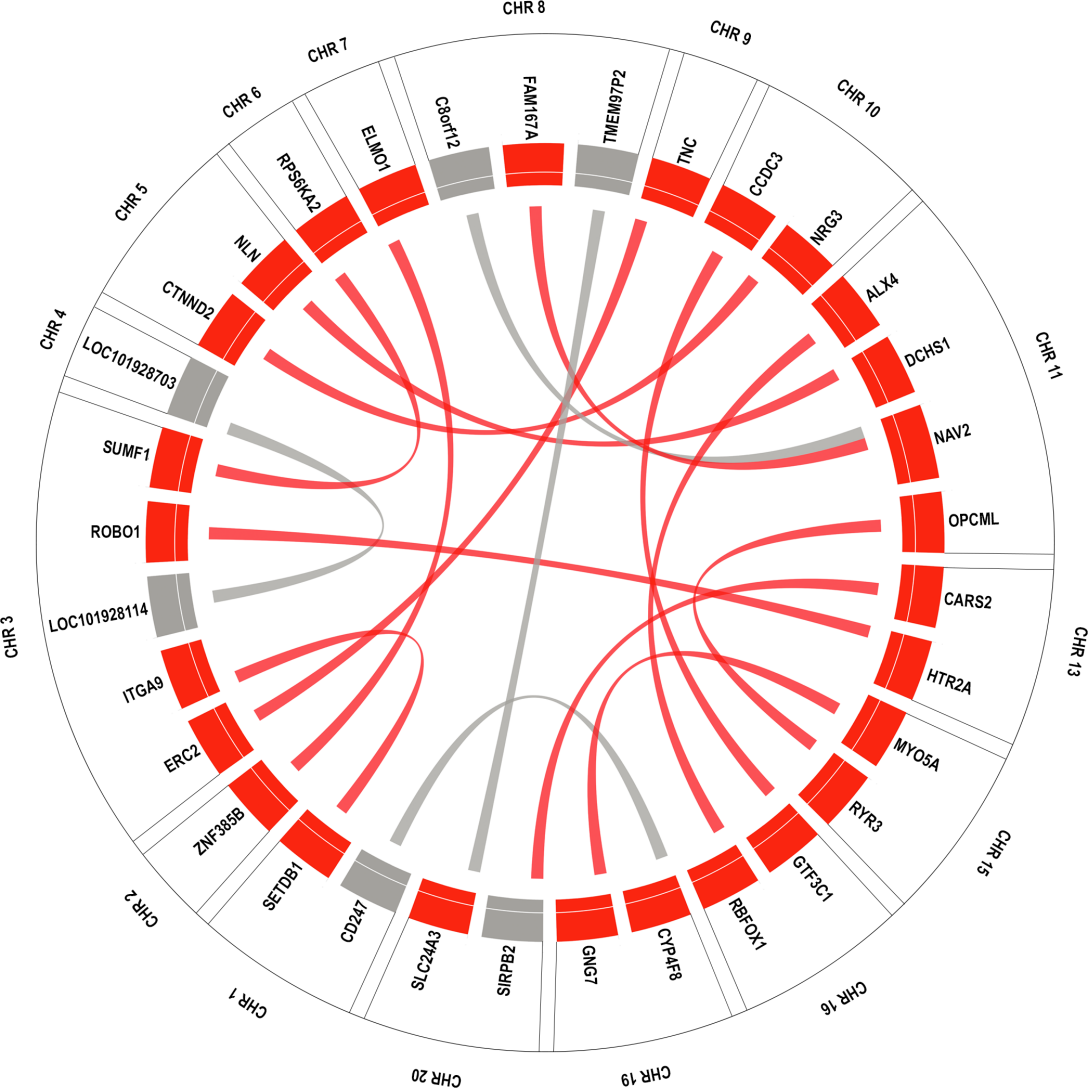
Circular plot representing only unique gene-gene interactions. Genes are colored as red if they are expressed in eye and as grey if not expressed in the eye. All genes are ordered by chromosome. Links between the genes represent gene-gene interactions that are replicated.

**Fig 5:**
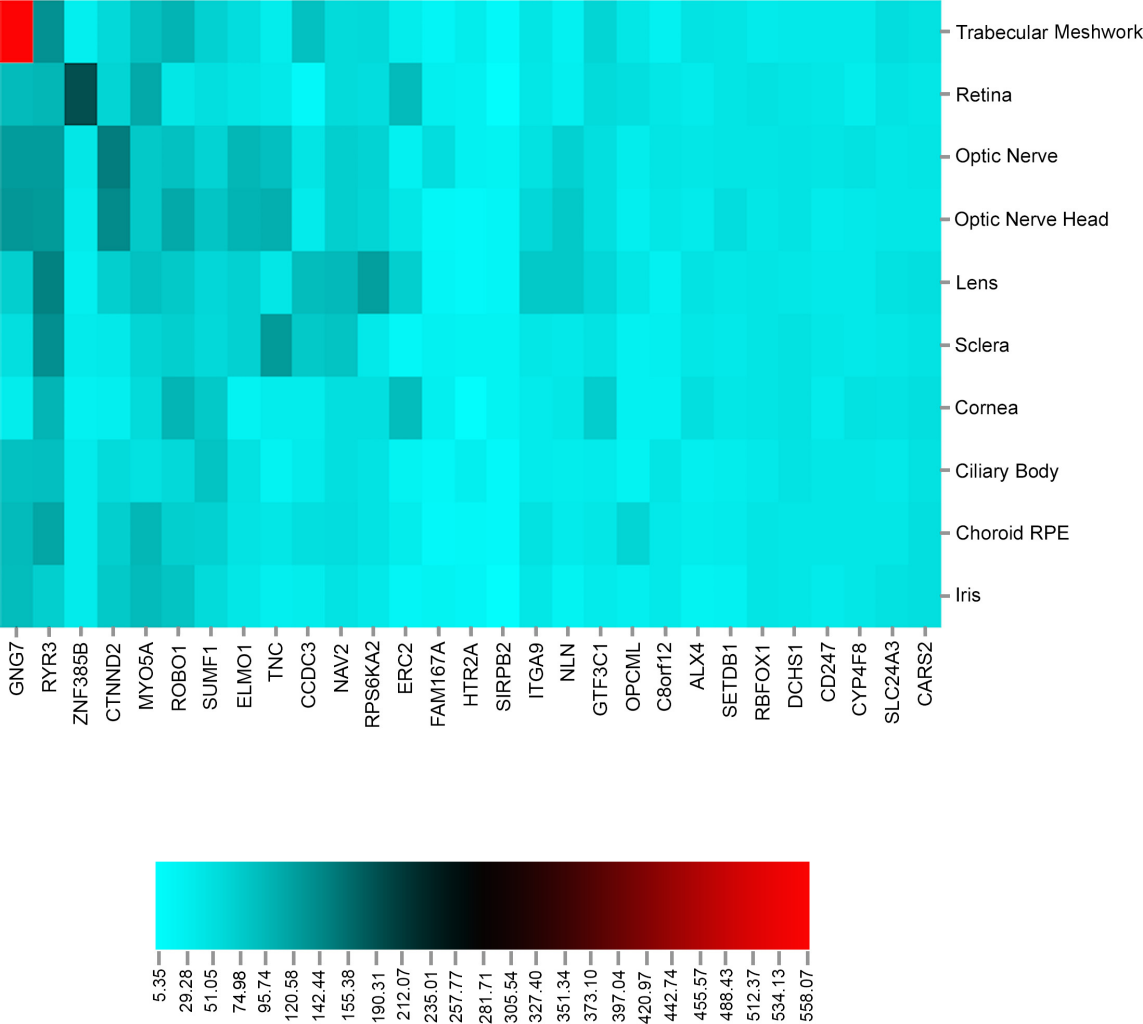
Heat map representing expression of the unique genes found in the ocular tissue database from GXG interaction results. Here, the x-axis lists all genes and y-axis lists the names of 10 tissues from the eye. Scale and color intensity represent PLIER values as reported in the ocular tissue database.

**Fig 6:**
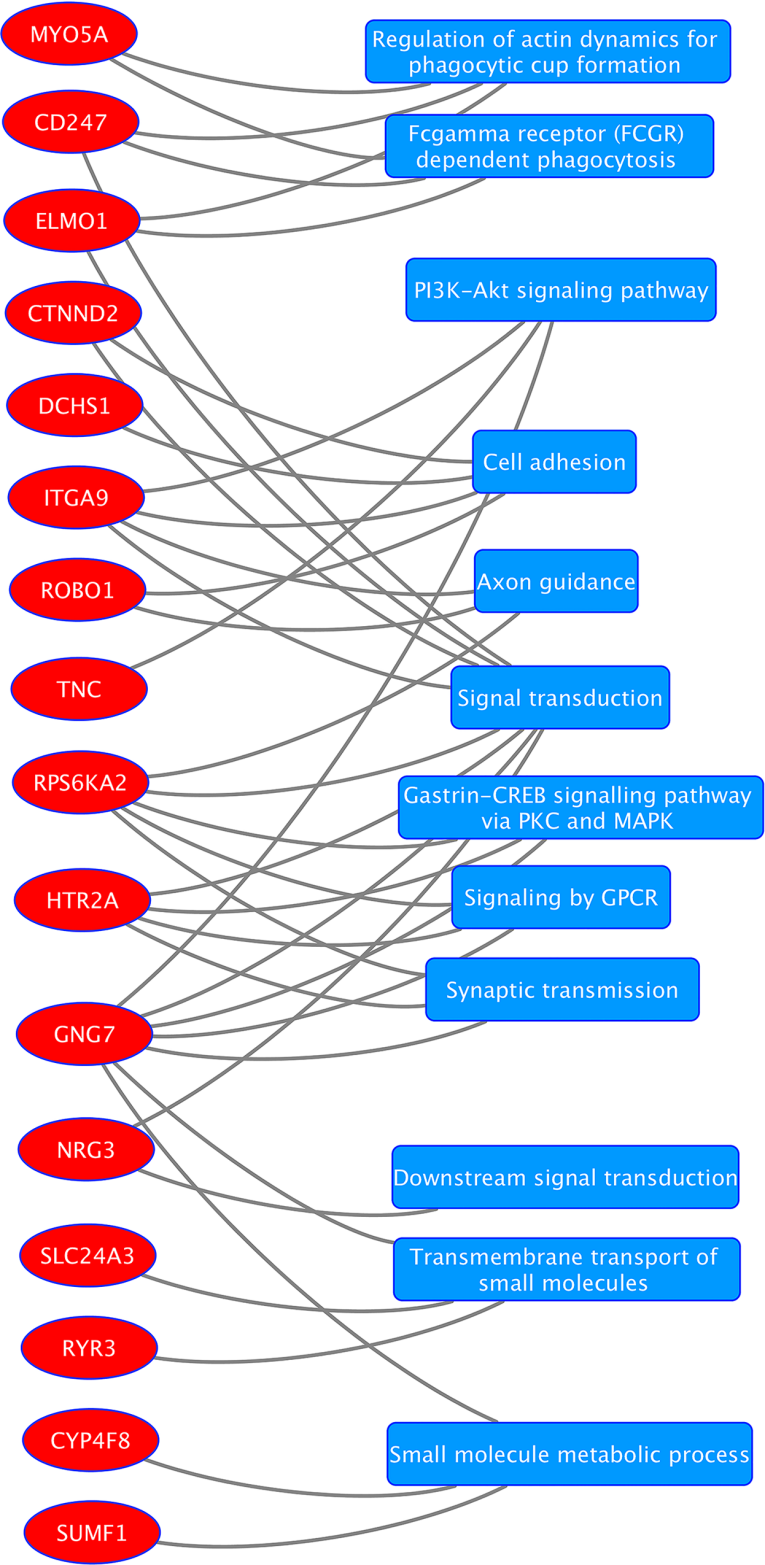
Hierarchal plot showing 16 genes in red circles and 11 pathways in blue rectangles. Connection between the genes and pathways was established using Biofilter 2.0.

As additional follow-up, we explored which of the 33 unique genes comprising the 17 unique gene-gene models (described above) showed evidence of expression in eye tissue using two online databases: Tissue-specific Gene Expression and Regulation (TiGER) and BioGPS[35]. **Fig 4**shows a gene-gene interaction circular plot among the 33 unique genes color-coded by the genes that are expressed (red) and not expressed (grey) in eye based on these databases. Next, we looked for expression of these genes in different eye tissues using the ocular tissue database[36] and reported the expression of the genes found in the database in 10 different eye tissues in **Fig 5.** We also annotated these 33 unique replicating genes to pathways using Biofilter 2.0. To evaluate the presence of genes in major pathways, only pathways supported by at least 2 sources in Biofilter were considered. Since Biofilter reports results from all the data sources, we observed some annotations whose relevance could not be established and these annotations were discarded. We carefully reviewed pathway annotations from Biofilter and removed results that were not applicable. **Fig 6**represents a Cytoscape hierarchal plot[37] for 16 of the 33 genes that were annotated to 11 major pathways.

Lastly, to investigate genetic variance explained by these SNPs, we combined 17 SNPs from Table 1 with the 34 SNPs from Table 2 to estimate variance of POAG risk. Analysis in eMERGE data using GCTA[38] suggests that GWAS SNPs explain 2.1% of the variance. Adding top significant SNPs from the interaction analysis performed here increases the explained estimated variance to 5.6%. Analysis in NEIGHBOR dataset for GWAS as well as SNPs replicated from interaction study explain 3.4% of the total variance. Variance explained in the NEIGHBOR dataset is less than the variance explained in eMERGE mainly because the disease prevalence (1.86% as suggested by National Eye Institute (https://nei.nih.gov/eyedata/glaucoma)) used in this analysis is low as compared to the proportion of cases and controls as suggested in GCTA manual[38].

**Table 2:** All replicating gene-gene interactions at LRT p-value <1e-04 for replication dataset.

Gene1_Gene2	eMERGE Var1	eMERGE Var2	eMERGE LRT P-val	Neighbor Var1	Neighbor Var2	Neighbor LRT P-val
**ALX4_RBFOX1**	rs10838251	rs653127	7.29E-06	rs7126447	rs11077011	1.62E-06
**OPCML_RYR3**	rs2246352	rs2467565	4.34E-06	rs2659614	rs8028442	1.01E-05
**ZNF385B_ELMO1**	rs2138196	rs918978	6.53E-06	rs13408749	rs2392477	1.23E-05
**CTNND2_NRG3**	rs1302802	rs11191761	9.23E-06	rs2973515	rs660464	1.60E-05
**TMEM97P2_SLC24A3**	rs7836543	rs7264540	9.24E-06	rs7826784	rs11907443	1.12E-04
**CD247_CYP4F8**	rs864537	rs2239367*	8.88E-06	rs704856	rs3794989	1.24E-04
**CCDC3_GTF3C1**	rs2399892	rs4787965	6.78E-06	rs2580904	rs7184872	1.91E-04
**ROBO1_HTR2A**	rs6802127	rs2070039	5.44E-06	rs1444480	rs9534510	2.18E-04
**ERC2_TNC**	rs7637114	rs7035322	4.88E-06	rs815443	rs7847271	2.42E-04
**CARS2_SIRPB2**	rs389656*	rs12480584	1.83E-06	rs2304767	rs2422575	3.83E-04
**FAM167A_NAV2**	rs7018324	rs10734289	6.59E-06	rs2618433	rs2584848	3.90E-04
**SUMF1_RPS6KA2**	rs11919486	rs12208871	2.39E-06	rs13096374	rs1883361	4.32E-04
**C8orf12_NAV2**	rs7018324	rs10734289	6.59E-06	rs10105588	rs2243437	6.09E-04
**SETDB1_ITGA9**	rs34207591	rs4678971	9.66E-06	rs198325	rs78196452	6.47E-04
**NLN_DCHS1**	rs10096	rs937856	2.97E-06	rs1309822	rs2659863	7.03E-04
**MYO5A_GNG7**	rs1669859	rs759065	3.01E-06	rs2693467	rs7258864	7.84E-04
**LOC101928114_LOC101928703**	rs12491137	rs6840100	9.30E-06	rs62248525	rs75134552	7.96E-04

## Discussion

We have successfully completed a discovery and replication study to test gene-based interactions in two large POAG datasets: eMERGE and the NEIGHBOR. We have identified several gene-gene interactions associated with glaucoma and showed that most of the genes are expressed in the eye. We annotated all replicating genes from the gene-gene interaction analysis to pathways and observed that 16 genes from the analysis were mapped to 11 major pathways based on the sources available in Biofilter.

Our results show several genes involved in cell adhesion, axonal guidance and signaling pathways, previously hypothesized to impact glaucoma-related optic nerve degeneration [39]. Expression analysis from the ocular tissue database also shows that many genes identified by this analysis show high expression in optic nerve and optic nerve head especially three genes in particular *GNG7*, *RYR3 and CTNND2* (See **Fig 5**).

Zinc finger proteins are also known to be associated with glaucoma[40]. Our results indicate associations with POAG involving interactions between several zinc finger proteins and other protein coding genes such as interaction between genes *ZNF385B* and *ELMO1* (LRT p-value 6.53–06 and 1.23E-05 for eMERGE and NEIGHBOR respectively).

Among the top associations, is the interaction between protein coding genes *OPCML* and *RYR3* (LRT p-value 4.34E-06 and 1.01E-05 for eMERGE and NEIGHBOR respectively). The *OPCML* gene is an opioid binding protein/cell-adhesion molecule that is closely related to other proteins including, the *NTN* (neurotrimin), and genes in the immunoglobin family (IgLON family)[41]. *NTN* has been found to be nominally associated with glaucoma in previous studies[42].

**Fig 7:**
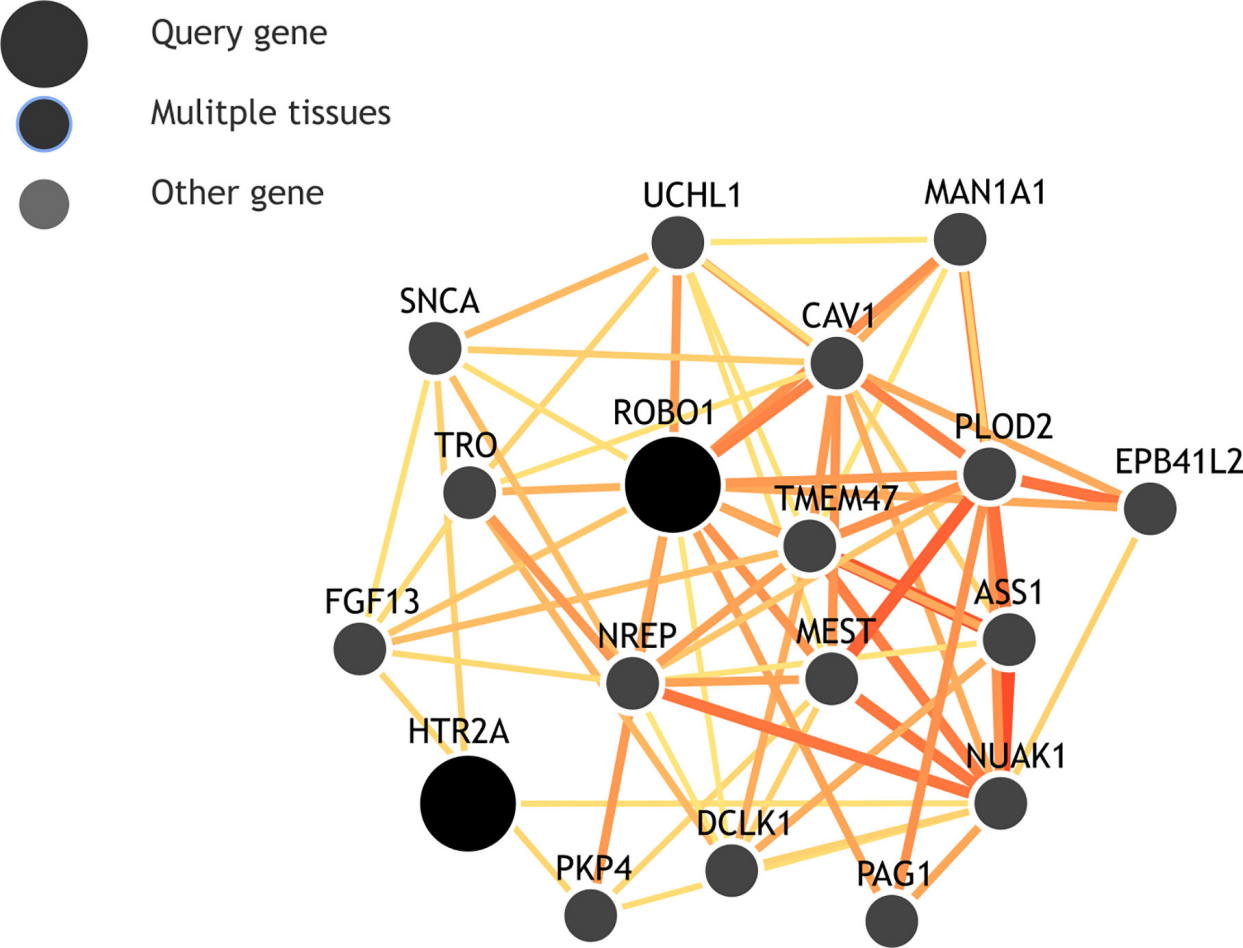
GIANT network representing interaction between *ROBO1* and *HTR2A* query genes and other genes for expression in the eye. Scale represents the confidence in the relationship shown.

Our results also indicate interaction for *ROBO1* and *HTR2A* (LRT p-values 5.44–06 and 2.18–04 for eMERGE and NEIGHBOR respectively as shown in **Table 2**). Both of these genes show co-expression with *SNCA* as predicted by the GIANT network [43] (Genome-scale Integrated Analysis of gene Networks in Tissues: http://giant.princeton.edu/) web based interface. **Fig 7**shows an interaction network generated by GIANT with genes that show functional interaction between query genes and other genes expressed only in the eye. *HTR2A* encodes receptors of 5-HT2A serotonin molecules. Serotonin molecules work as neurotransmitters and these molecules also function as receptors for many drugs[44].

Another interesting finding is an interaction between *CTNND2* and *NRG3* (LRT p-value 9.23E-06 and 1.60E-05 for eMERGE and NEIGHBOR respectively as shown in **Table 2**). *CTNND2*, i.e. Catenin Delta 2 gene, encodes adhesive junction proteins that are involved in retinal morphogenesis and cell adhesion [45,46].

Cadherin molecules are known to support the migration of axons during optic nerve morphogenesis [47][50] and have been previously investigated as POAG susceptibility genes [48,49]. Our results identify a statistical interaction between *NLN* and glycoprotein DCHS1 also known as *PCDH7* (LRT p-value 2.97E-06 and 7.035E-04 for eMERGE and the NEIGHBOR respectively) and also an interaction between *NGR3* (neuregulin 3) and cadherin-associated protein *CTNND2* (LRT p-value 9.23E-06 and 1.60E-05 for eMERGE and the NEIGHBOR respectively).

Pathway analysis using the gene-gene models revealed several interesting pathways including signal transduction, cell adhesion and regulation of actin dynamics for phagocytic cup formation. Myocilin, a protein involved in mendelian forms of glaucoma, may impact focal adhesion and migration of cells [50] but the interaction among other genes with similar functions is unknown. Our analysis highlights several genes with these functions and their role in glaucoma may require further investigation.

Our interaction analysis showed at least two genes (*CTNND2* and *RBFOX1*) that have been previously associated with myopia, and are also associated with glaucoma in our datasets. *CTNND2* is known to be associated with myopia[51] and myopia can influence glaucoma risk [52]. *CTNND2* is significantly associated with myopia in an Asian GWAS and *RBFOX1* has been associated with myopia in a multi-ethnic cohort and also in a Chinese population-based study [53–55]. In eMERGE, POAG cases and controls were not screened for myopia. Therefore, these associations will need further evaluation to understand if they are due to the co-morbidities of POAG and myopia in these patients. These results suggest that exploring interaction among genes associated with myopia may also be of interest in POAG studies. In conclusion, interactions among genes with only marginal POAG association can demonstrate interesting interactions that may contribute to the complex etiology of POAG.

This is the first replication study to identify gene-gene interactions in POAG in a large case-control dataset. We found 17 gene-gene replicating models at LRT p-value less than 0.001 and many of these interacting genes play important roles in cell adherence junction and signal transduction. Most drugs for glaucoma target synthesis of aqueous humor, which could be achieved by manipulating trabecular meshwork that maintains the outflow of aqueous humor in the eye. In most of the glaucoma patients, the outflow mechanism from the trabecular meshwork is negotiated when the progression of the disease is accompanied by an increase in intraocular pressure. For all the genes that are expressed in eye, we looked at the genes that are also expressed in trabecular meshwork via literature search and in the ocular tissue database as shown in **Fig 5**[53,54]. While most of the genes identified in this study show significant expression in the eye, 9 genes in particular (*GNG7*, *ROBO1*, *SUMF1*, *RYR3*, *SLC24A3*, *CCDC3*, *CARS2*, *RPS6KA*, *SETDB1*) also show expression in trabecular meshwork. Thus, this suggests potential genes that could be involved in ocular hypertension and elevated IOP could be linked to the progression of POAG. Many complex diseases are caused by multiple variants in the same genes or in different genes. SNP-based association tests do not account for locus based genetic heterogeneity, and when looking for replication it is highly unlikely that the same variant might be causal for a complex disease in an independent population. Our analysis highlights the importance of performing gene based interaction analysis to utilize complexity in biology and considering heterogeneity for testing statistical epistasis. Our analysis also indicates that testing for interactions in association studies can help explain additional genetic variance beyond that accounted for by SNPs associated with a disease via main effects only. Despite of the advantages to look for gene-based interactions, one limitation of this approach is that focusing only on the genes could result in missing interactions among the intragenic regulatory regions such as distal enhancers. Furthermore, we also did not test for all possible pairwise combinations but instead picked variants that show significant to marginal main effect which could also result in missing some potentially significant epistatic variants. Future studies could include testing for interactions based on prior-biological knowledge and testing for regions that are involved in similar pathways or are linked based on regulatory information available[32]. More studies or meta-analysis to replicate the interaction results can provide increased confidence in our analyses.

In conclusion, we identified many significant gene-gene models that are associated with POAG and our study clearly indicates that we can consider heterogeneity among independent populations by looking for replication in gene regions rather than exact SNPs. Thus presenting a gene based SNP-SNP interaction analysis, which has been done by others including Ma et al. 2012 and 2013[56,57], as a powerful approach in replication studies.

## Materials and Methods

### Discovery dataset

**Fig 8:**
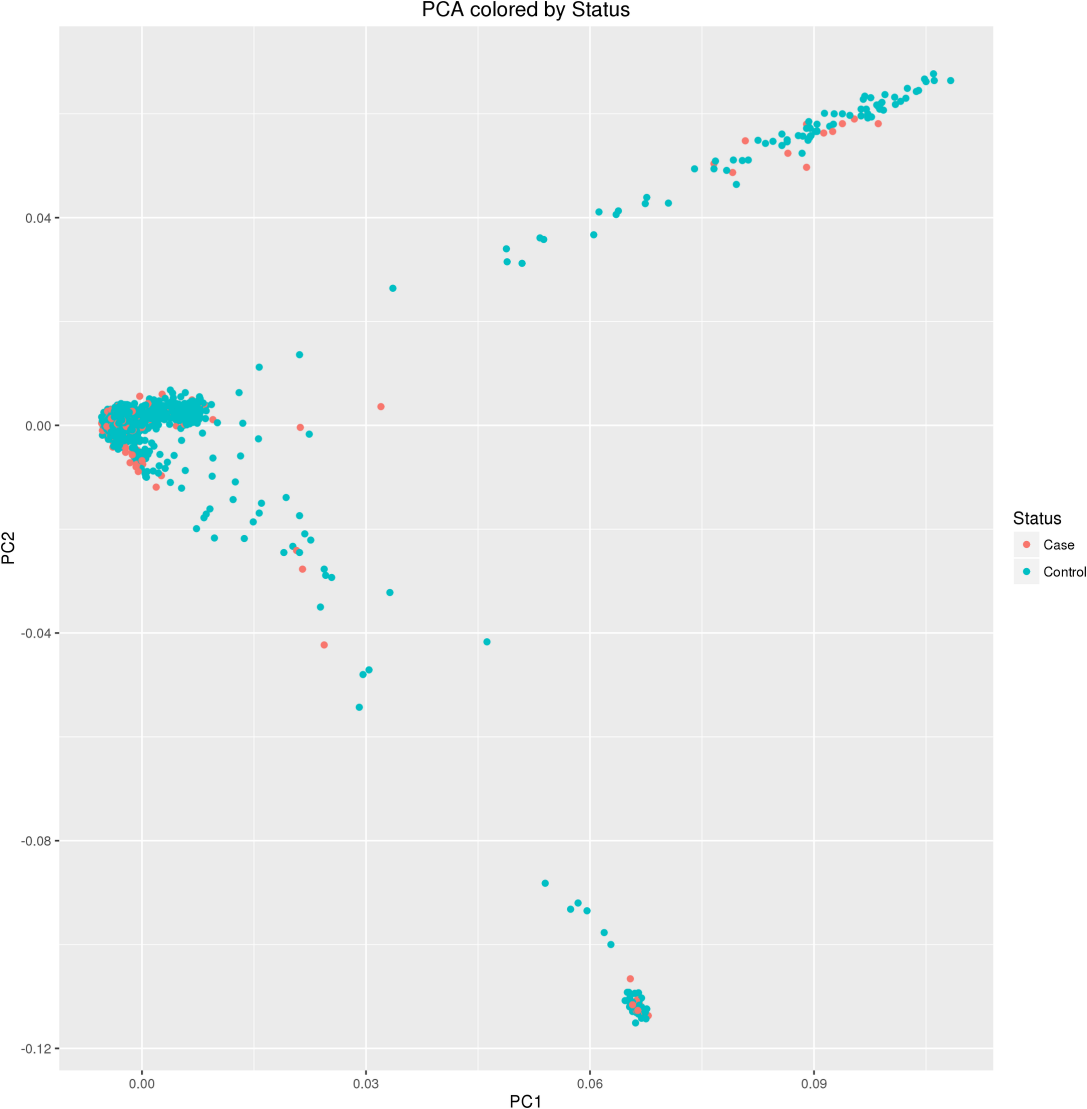
Principal component analysis (PCA) of 5090 samples (glaucoma cases and controls) from eMERGE network. Eigenvector 1 is on x-axis and eigenvector 2 is on y-axis. Each point is a sample and they are color coded by case and control status.

eMERGE-II is a consortium in the United States that consists of seven adult sites and two pediatric sites across the country. There are a total of 55,289 unique samples in eMERGE that have been genotyped on different platforms and imputed to the 1000 Genomes March 2012 reference dataset using SHAPEIT2 for phasing and IMPUTE2 for imputation [13]. Five adult sites (Marshfield Clinic, Group Health, Geisinger Clinic, Northwestern University, and Mayo Clinic) contributed samples to the glaucoma phenotype. There are a total of 5090 samples that include 961 cases and 4,129 controls, (2,247 males and 2,843 females). **Table 3**shows the distribution of samples across all sites and gender. Glaucoma case and control status was obtained from EMR data. All cases and controls selected were age 40 or above and had a recent eye exam within 2 years. Patients with two or more occurrences for ICD-9 codes for open angle (365.10), primary open angle (365.11) or low-tension open angle glaucoma (365.12) were diagnosed as cases and all the other samples were further evaluated for controls by following criteria: excluded any samples that had diagnosis for Ocular Hypertension; patients with Current Procedural Terminology (CPT) codes for glaucoma related surgery; patients that were given intraocular pressure lowering medications (details in supplementary S1 Text). Glaucoma samples in the eMERGE dataset were extracted from the imputed version3 dataset (dbGaP accession: phs000888.v1.p1}; all markers and samples below 99% call rate were excluded from the analysis. For the purpose of running GWAS and GXG common variant analysis, we only kept markers at MAF > = 0.05. This resulted in 2,914,185 SNPs tested for GWAS. Principal component analysis was performed using Eigensoft [58] on all non-related samples to look for individual level population variations. A plot of eigenvector 1 versus 2 from the eigen-analysis is shown in **Fig 8**. Adjusting for principal components helps to identify non-spurious and relevant associations that are merely due to ancestral difference.

**Table 3:** Demographics for glaucoma samples in eMERGE discovery dataset.

Variable	Type	Category	Observations	%
**Glaucoma Phenotype**	**Cases**		961	0.189
	**Controls**		4129	0.811
**Site**	**Cases**	Group Health	409	0.080
		Vanderbilt	71	0.014
		Marshfield	289	0.057
		Mayo	86	0.017
		Geisinger	85	0.017
		Northwestern	21	0.004
	**Controls**	Group Health	1441	0.283
		Vanderbilt	253	0.050
		Marshfield	1245	0.245
		Mayo	411	0.081
		Geisinger	382	0.075
		Northwestern	397	0.078
**Sex**	**Cases**	Male	404	0.079
		Female	557	0.109
	**Controls**	Male	1843	0.362
		Female	2286	0.449
**Race**	**Cases**	Black	14	0.003
		White	922	0.181
		Other	25	0.005
	**Controls**	Black	82	0.016
		White	3918	0.770
		Other	129	0.025

### Replication dataset

DNA from more than 4,900 POAG cases and controls (primarily European Americans) was collected for the NEIGHBOR study from 12 sites including Massachusetts Eye and Ear Infirmary, Brigham and Women’s Hospital, Duke University, Johns Hopkins University, Marshfield Clinic, Stanford University, University of Pittsburgh, University of West Virginia, University of Miami, University of Michigan, University of California, San Diego, and Vanderbilt University as previously reported[6,29]. POAG was defined in cases by the presence of optic nerve disease with visual field loss consistent with nerve fiber layer dropout; when this information was not available, cases were determined based on a cup-to-disc ratio greater than 0.8 in at least one eye. POAG controls had normal optic nerves, normal intraocular pressure, and presumed normal visual fields, or a cup to disc ratio less than 0.7. Samples were genotyped on the Illumina 660W Quad platform at the Center for Inherited Diseases Research (CIDR). Samples with call rate <97% were excluded, as were samples determined to be related with a kinship coefficient >0.0312. This resulted in 4,422 individuals (2,132 POAG cases, 2,290 controls). Principal components analysis was run using Eigenstrat and eigenvectors were tested for association with disease status using a logistic regression model in Stata; eigenvectors 1,2,3, and 10 were significant. The GWAS data were phased and imputed to the March 2012 version of the 1000Genomes reference data using ShapeIt2 and IMPUTE2. Markers with call rate <97% and MAF<0.05 were removed, as were markers with imputation quality <0.7; this resulted in 6,287,101 variants to be examined for further analysis. Out of ~6M variants, 3839 SNPs were used for the purpose of replication as described earlier in methods section.

### SNP-SNP interaction analysis

Exploring pairwise SNP-SNP interaction is a two-step process. Steps for filtering of SNPs at different stages are explained in detail in **Fig 2**. From GWAS results, we extracted all SNPs with main effect p-value <0.01 and also to test only independent models, we removed any SNPs in high linkage disequilibrium (LD r2 threshold >0.6). We removed these SNPs in LD using PLINK. Secondly, we investigated all pairwise SNP-SNP interaction models using logistic regression in PLATO (https://ritchielab.psu.edu/software/plato-download) assuming an additive encoding model. All models were adjusted for age, sex, site, platform and first 6 principal components. Likelihood ratio tests were performed to determine significance of each pairwise interaction model above and beyond its main effects. This resulted in 117 SNP-SNP models with LRT p-value <1e-05. To perform replication, these SNPs from the top 117 SNP-SNP models were annotated to genes using Biofilter 2.0[59] which resulted in 91 unique genes that were considered for replication. All SNPs in these genes were extracted from NEIGHBOR data and then SNPs passing an info score threshold of 0.7, marker call rate of 99%, MAF 0.05 and SNPs passing LD pruned r^2^ threshold 0.6 were considered for replication (i.e. 3,839 SNPs). All genes based on expanded SNP-SNP models were exhaustively tested in the replication dataset. A total of 7,367,042 pairwise SNP-SNP models (from 3,839 SNPs) in 91 genes were evaluated in the replication dataset. Unique models with replicating LRT p-value< 0.001 are reported in Table 2.

### Ethics statement

All eMERGE and NEIGHBOR consortium sites have appropriate IRB approvals for this de-identified research. All relevant data are within the paper and its Supporting Information files. Raw genotype files for eMERGE (phs000888.v1.p1) and NEIGHBOR (phs000238.v1.p1) are at dbGaP.

## Supporting Information

S1 Text Phenotypic algorithm for POAG from eMERGE. Flowchart as well as pesudocode for phenotypic algorithm to extract samples from Electronic Health Record data to classify as cases and controls for POAG.
(DOC)

S1 Table Power calculation results. Power calculation as performed by Quanto considering odds ratio in range of 1–3. Two separate sheets for disease risk of 0.0001 and 0.018
(XLSX)

S2 Table All Replicated results between eMERGE and NEIGHBOR datasets. All SNP-SNP interaction results replicated in eMERGE and NEIGHBOR analysis. This table lists p-values for all eMERGE samples analysis and also analysis in European American samples only. P-values for each variable in the model as well as likelihood ratio test p-value for interactive effect is also provided.
(XLSX)

S1 Fig Quantile-Quantile plot of interaction results from discovery (eMERGE) data. QQ-plot showing distribution of p-values from interaction analyses in discovery dataset.
(TIFF)
