# Supplementary material for: Epistatic Gene-Based Interaction Analyses for Glaucoma in eMERGE and NEIGHBOR Consortium
Source: PLoS Genet. 2016 Sep 13;12(9):e1006186. doi: 10.1371/journal.pgen.1006186 (PMC5021356; doi:10.1371/journal.pgen.1006186)
Supplement: S1 Text — Flowchart as well as pesudocode for phenotypic algorithm to extract samples from Electronic Health Record data to classify as cases and controls for POAG. (DOC) [file pgen.1006186.s001.doc]

**Genome-Wide Study of Glaucoma**

**in the Personalized Medicine Research Project**

Below is a flowchart and pseudo code used to select Marshfield’s Glaucoma cohort. Pseudo code can be found on the following pages. Included is information specific to each reference identified within the applicable flowchart symbols.

If you have questions regarding any of the information presented on this page, you may contact either:

Peggy Peissig at [peissig.peggy@marshfieldclinic.org](mailto:peissig.peggy@marshfieldclinic.org) or call: 715.221.9880

James Linneman at [linneman.james@marshfieldclinic.org](mailto:linneman.james@marshfieldclinic.org) or call 715.221.7271

**FLOWCHART of Glaucoma Phenotyping Process**

**Pseudo code for the “Glaucoma” Phenotype**

| **ID** | **Step** | **Description** |
| --- | --- | --- |
|  |  |  |
| 1 | a | -Select all subjects from the PMRP cohort who have:   - Consented - Did not withdraw from the study - Include subjects with contact_for_research = ‘N’ - Include subjects where questionnaires have been scanned. |
| 2 | a | -Exclude subjects with the following ICD9 codes:  - Childhood Glaucoma 365.14  - Glaucoma associated with ocular trauma 365.65 |
| 3 | a | -Select subjects with the following diagnoses codes:  -Use diagnoses from 1960 to present. |
|  |  | Diagnoses of any glaucoma  'ICD 9' - BETWEEN '365.00' AND '365.03'  'ICD 9' - BETWEEN '365.10' AND '365.13'  'ICD 9' - BETWEEN '365.20' AND '365.9'  'HICDA 2' - BETWEEN '375.0' AND '375.2'  'HICDA 2' - = '375.9'  'HICDA 1' - BETWEEN '375.0' AND '375.9'  'ICDA 8' - ='387.0' |
|  |  |  |
| 4 | a | -For subjects having no glaucoma diagnoses (see 3a above), select subjects having none of the following diagnoses codes: |
|  |  | Diagnoses of ocular hypertension |
|  |  | 'ICD 9' - = '365.04'  'HICDA 2' - = '375.3'  'HICDA 1' - = '378.909' |
| 5 | a | -For subjects having no Ocular Hypertension diagnoses (see 4a above), select subjects having no intraocular pressure lowering medications where route of administration is ophthalmic. Intraocular pressure lowering medications:  Where THERAPEUTIC_SPECIFIC_DESC='Miotics/Other Intraoc. Pressure Reducers' AND DRUG_ROUTE_DESC='Ophthalmic' |
|  |  | (See pages 4-5 for complete list). |
|  |  |  |
|  |  |  |
| 6 | a | -Select from subjects having none of the following glaucoma type surgery procedures:  -Surgeries to increase drainage of fluid from eye (for open-angle or chronic closed-angle glaucoma):  Trabeculectomy 66170 66172  Tube-shunt surgery (seton glaucoma) 66180 66185  Laser trabeculoplasty 65855  Laser sclerostomy 66710 66711  Other Glaucoma surgeries:  Surgery to prevent closure of the drainage angle:  Surgical iridectomy 66625 66630  Laser iridotomy 66761 |
|  |  | **Pseudo code for the “Glaucoma” Phenotype** |
| **ID** | **Step** | **Description** |
|  |  |  |
| 6 | a | Surgery to decrease the amount of fluid produced in the eye:  Cyclocryopathy 66720  Laser cyclophotocoagulation 66710 66711  Surgery for congenital glaucoma (in children):  Goniotomy 65820  Trabeculectomy 65850 |
| 7 | a | -Select from subjects having at least one eye exam within the last two years if living or the last two years prior to date of death if deceased, using CPT codes: 92002, 92004, 92012, 92014, 92018, 92019,  or 99201, 99202, 99203, 99204, 99205, 99211, 99212, 99213, 99214, 99215, 99241, 99242, 99243, 99244, 99245 where provider specialty is Ophthalmology or Optometry. |
|  |  |  |
| 8 | a | -Select from subjects age 50 years or older at most recent eye exam (within last two years). |
|  |  |  |
| 9 | a | -Select from subjects having one or more comprehensive type eye exams (ever) using CPT codes: 92004, 92014,  or 99204, 99205, 99214, 99215, 99244, 99245 where provider specialty is Ophthalmology or Optometry. |
|  |  |  |
| 10 | a | -Select from subjects having the following Open-angle glaucoma ICD9 codes:  Open-angle glaucoma (unspecified) 365.10  Primary open-angle glaucoma 365.11  Low-tension open-angle glaucoma 365.12 |
| 11 | a | -Select from subjects having Open-angle glaucoma ICD9 codes given by ophthalmologist/optometrist within ophthalmology/optometry department(s). |
|  |  |  |
| 12 | a | -Select subjects having two or more open-angle glaucoma diagnoses (see 10a above); within ophthalmology/optometry (see 11a above). |
|  |  |  |
| 13 | a | -Select subjects having two or more open-angle glaucoma diagnoses (see 10a above) within ophthalmology/optometry (see 11a above), where the earliest and most recent diagnosis are at least fourteen days apart. |
|  |  |  |
| 14 | a | -Select from subjects having two or more open-angle glaucoma diagnoses (see 10a above) within ophthalmology/optometry (see 11a above), where the earliest and most recent diagnosis are at least fourteen days apart, and the subjects age at earliest open-angle glaucoma diagnosis is 40 years or older. |

|  |  | **Pseudo code for the “Glaucoma” Phenotype** |
| --- | --- | --- |
| **ID** | **Step** | **Description** |

Intraocular pressure lowering medications (where route of administration is ophthalmic):

| AHFS_SPECIFIC_CATEGORY_DESC | GENERIC_NAME | DRUG_NAME |
| --- | --- | --- |
| Alpha-Adrenergic Agonists (Eent) | BRIMONIDINE TARTRATE | ALPHAGAN |
| Alpha-Adrenergic Agonists (Eent) | BRIMONIDINE TARTRATE | ALPHAGAN P |
| Alpha-Adrenergic Agonists (Eent) | BRIMONIDINE TARTRATE | BRIMONIDINE TARTRATE |
| Alpha-Adrenergic Agonists (Eent) | BRIMONIDINE TARTRATE/TIMOLOL | COMBIGAN |
| Beta-Adrenergic Blocking Agents (Eent) | BETAXOLOL HCL | BETAXOLOL HCL |
| Beta-Adrenergic Blocking Agents (Eent) | BETAXOLOL HCL | BETOPTIC |
| Beta-Adrenergic Blocking Agents (Eent) | BETAXOLOL HCL | BETOPTIC S |
| Beta-Adrenergic Blocking Agents (Eent) | LEVOBUNOLOL HCL | AKBETA |
| Beta-Adrenergic Blocking Agents (Eent) | LEVOBUNOLOL HCL | BETAGAN |
| Beta-Adrenergic Blocking Agents (Eent) | LEVOBUNOLOL HCL | LEVOBUNOLOL HCL |
| Beta-Adrenergic Blocking Agents (Eent) | METIPRANOLOL | METIPRANOLOL |
| Beta-Adrenergic Blocking Agents (Eent) | METIPRANOLOL | OPTIPRANOLOL |
| Beta-Adrenergic Blocking Agents (Eent) | TIMOLOL | BETIMOL |
| Beta-Adrenergic Blocking Agents (Eent) | TIMOLOL MALEATE | ISTALOL |
| Beta-Adrenergic Blocking Agents (Eent) | TIMOLOL MALEATE | TIMOLOL MALEATE |
| Beta-Adrenergic Blocking Agents (Eent) | TIMOLOL MALEATE | TIMOPTIC |
| Beta-Adrenergic Blocking Agents (Eent) | TIMOLOL MALEATE | TIMOPTIC-XE |
| Beta-Adrenergic Blocking Agents (Eent) | TIMOLOL MALEATE/PF | TIMOPTIC |
| Beta-Adrenergic Blocking Agents (Eent) | TIMOLOL MALEATE/PF | TIMOPTIC OCUDOSE |
| Carbonic Anhydrase Inhibitors (Eent) | BRINZOLAMIDE | AZOPT |
| Carbonic Anhydrase Inhibitors (Eent) | DORZOLAMIDE HCL | DORZOLAMIDE HCL |
| Carbonic Anhydrase Inhibitors (Eent) | DORZOLAMIDE HCL | TRUSOPT |
| Carbonic Anhydrase Inhibitors (Eent) | DORZOLAMIDE HCL/TIMOLOL MALEAT | COSOPT |
| Carbonic Anhydrase Inhibitors (Eent) | DORZOLAMIDE HCL/TIMOLOL MALEAT | DORZOLAMIDE-TIMOLOL |
| Eent Drugs, Miscellaneous | APRACLONIDINE HCL | APRACLONIDINE HCL |
| Eent Drugs, Miscellaneous | APRACLONIDINE HCL | IOPIDINE |
| Eent Drugs, Miscellaneous | CARTEOLOL HCL | CARTEOLOL HCL |
| Eent Drugs, Miscellaneous | CARTEOLOL HCL | OCUPRESS |
| Miotics | CARBACHOL | CARBACHOL |
| Miotics | CARBACHOL | CARBASTORZ |
| Miotics | CARBACHOL | CARBOPTIC |
| Miotics | CARBACHOL | ISOPTO CARBACHOL |
| Miotics | DEMECARIUM BROMIDE | HUMORSOL |
| Miotics | ECHOTHIOPHATE IODIDE | PHOSPHOLINE IODIDE |
| Miotics | PHYSOSTIGMINE SULFATE | ESERINE SULFATE |
| Miotics | PHYSOSTIGMINE SULFATE | PHYSOSTIGMINE SULFATE |
| Miotics | PILOCARPINE HCL | ADSORBOCARPINE |
| Miotics | PILOCARPINE HCL | AKARPINE |
| Miotics | PILOCARPINE HCL | ALMOCARPINE |

|  |  | **Pseudo code for the “Glaucoma” Phenotype** |
| --- | --- | --- |
| **ID** | **Step** | **Description** |

| Miotics | PILOCARPINE HCL | INFA-KARPINE |
| --- | --- | --- |
| Miotics | PILOCARPINE HCL | I-PILOPINE |
| Miotics | PILOCARPINE HCL | ISOPTO CARPINE |
| Miotics | PILOCARPINE HCL | OCUSERT PILO-20 |
| Miotics | PILOCARPINE HCL | OCUSERT PILO-40 |
| Miotics | PILOCARPINE HCL | PILOCAR |
| Miotics | PILOCARPINE HCL | PILOCARPINE HCL |
| Miotics | PILOCARPINE HCL | PILOKAIR |
| Miotics | PILOCARPINE HCL | PILOMIOTIN |
| Miotics | PILOCARPINE HCL | PILOPINE HS |
| Miotics | PILOCARPINE HCL | PILOPTIC-1 |
| Miotics | PILOCARPINE HCL | PILOPTIC-2 |
| Miotics | PILOCARPINE HCL | PILOPTIC-3 |
| Miotics | PILOCARPINE HCL | PILOPTIC-4 |
| Miotics | PILOCARPINE HCL | PILOPTIC-6 |
| Miotics | PILOCARPINE HCL | PILOSOL |
| Miotics | PILOCARPINE HCL | PILOSTAT |
| Miotics | PILOCARPINE HCL | SPECTRO-PILO |
| Miotics | PILOCARPINE HCL | STORZINE 1 |
| Miotics | PILOCARPINE HCL | STORZINE 2 |
| Miotics | PILOCARPINE HCL | STORZINE 4 |
| Miotics | PILOCARPINE HCL/EPI BIT | E-PILO-1 |
| Miotics | PILOCARPINE HCL/EPI BIT | E-PILO-2 |
| Miotics | PILOCARPINE HCL/EPI BIT | E-PILO-3 |
| Miotics | PILOCARPINE HCL/EPI BIT | E-PILO-4 |
| Miotics | PILOCARPINE HCL/EPI BIT | E-PILO-6 |
| Miotics | PILOCARPINE HCL/EPI BIT | P1E1 |
| Miotics | PILOCARPINE HCL/EPI BIT | P2E1 |
| Miotics | PILOCARPINE HCL/EPI BIT | P3E1 |
| Miotics | PILOCARPINE HCL/EPI BIT | P4E1 |
| Miotics | PILOCARPINE HCL/EPI BIT | P6E1 |
| Miotics | PILOCARPINE NITRATE | PILAGAN |
| Miotics | PILOCARPINE NITRATE | PV CARPINE |
| Prostaglandin Analogs | BIMATOPROST | LUMIGAN |
| Prostaglandin Analogs | LATANOPROST | LATANOPROST |
| Prostaglandin Analogs | LATANOPROST | XALATAN |
| Prostaglandin Analogs | TRAVOPROST | TRAVATAN Z |
| Prostaglandin Analogs | TRAVOPROST (BENZALKONIUM) | TRAVATAN |
| Prostaglandin Analogs | UNOPROSTONE ISOPROPYL | RESCULA |

Last updated: April 25, 2012
