# Supplementary figures and images for: Epistatic Gene-Based Interaction Analyses for Glaucoma in eMERGE and NEIGHBOR Consortium
Source: PLoS Genet. 2016 Sep 13;12(9):e1006186. doi: 10.1371/journal.pgen.1006186 (PMC5021356; doi:10.1371/journal.pgen.1006186)

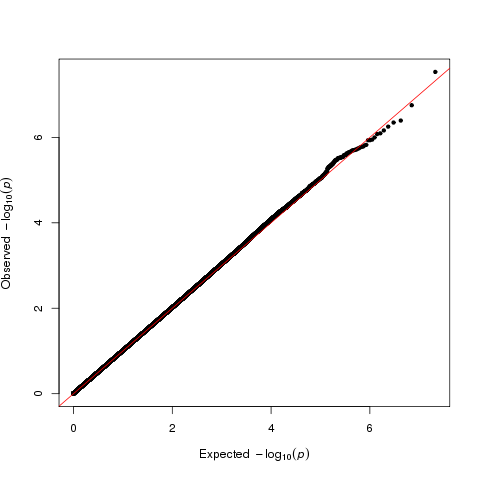

Supplement: S1 Fig — QQ-plot showing distribution of p-values from interaction analyses in discovery dataset. (TIFF) [file pgen.1006186.s004.tiff]
